# Important bioactives carnitine and betaine in milk and dairy products: a systematic review based on text mining and topic analysis

**DOI:** 10.1007/s44463-026-00112-6

**Published:** 2026-10-01

**Authors:** Lucia Trapanese, Angela Salzano, Nunzio Antonio Cacciola, Gianluca Neglia, Giovanna Bifulco, Marialuisa Balestrieri, Michael J. D´Occhio, Giuseppe Campanile

**Affiliations:** 1 Department of Veterinary Medicine and Animal Production, University of Naples Federico II, Naples, Italy; 2 Department of Precision Medicine, University of Campania Luigi Vanvitelli, Naples, Italy; 3 School of Life and Environmental Sciences, Faculty of Science, The University of Sydney, Sydney, Australia

**Keywords:** Dairy, Text mining, Topic analysis, Biomolecules, Health

## Abstract

A systematic review traced the evolution of research on carnitines and betaines in milk and dairy products. A bibliometric search of the Scopus^®^ database, using keywords such as “milk/dairy products and biomolecules”, “milk/dairy products and carnitine/betaine” retrieved 1.965 publications spanning January 1970 to December 2024. After screening, 179 records were selected for analysis. The most recurrent terms were “metabolit” “cow” and “infant”, highlighting the role of milk in the human diet. Topic analysis identified three main themes: Topic 1 grouped studies on metabolic regulation in ruminants and the diet-driven modulation of the milk metabolome; Topic 2 grouped studies on carnitine and betaine as conditionally essential nutrients in early-life nutrition and infant development; Topic 3 grouped studies on dairy bioactives as nutraceuticals in adult chronic disease prevention and therapy. This systematic review recognized the main research themes on the bioactive properties of carnitine and betaine and identified gaps in knowledge and areas for future basic and translational research on the functional role of these bioactives in human and animal health.

## Introduction

Milk and dairy products are an important source of essential nutrients such as calcium, fats, high-quality proteins, vitamins and minerals (Tunick and Hekken 2015). Milk also contains a wide variety of bioactive molecules that offer additional health benefits including support for energy metabolism, liver function and the prevention of metabolic disorders (Pezeshki et al. 2023). The bioactive properties of milk (Kępka et al. 2022) and other dairy products (Teren et al. 2020) places milk in the category of functional foods (Tidona et al. 2009). Among the bioactive compounds are casomorphins, casokinins, casoplatelin and lactoferrin (Suh 2022). Particularly notable biomolecules that are widely present in milk and dairy products belong to the betaine and carnitine families (Servillo et al. 2018). These families of compounds have an essential role in human health due to their antioxidant, anti-inflammatory and anticancer properties (D’Onofrio et al. 2019, 2021; Zhao et al. 2018). L-Carnitine is synthesized from the amino acids L-lysine and L-methionine and is involved in fatty acid metabolism (Hayden et al. 2017). Its primary function is to facilitate the transport of long-chain fatty acids across the mitochondrial membrane, where they undergo β-oxidation to generate energy (Antunes et al. 2022). Betaine refers to a family of molecules produced through choline metabolism or obtained from food or supplements (Craig 2004; Willingham et al. 2020). These molecules serve as important osmolytes and are a major source of methyl groups. Betaines exhibit antioxidant, antitumor, anti-inflammatory, antimicrobial, antidiabetic and antiviral activities (Tidona et al. 2009).

The important roles of betaine and carnitine make these bioactives ideal candidates for a systematic review of the relevant literature. To accumulate information for the present review, we employed Text Mining (TM) and Topic Analysis (TA). These are two computational techniques based on Machine Learning (ML) that systematically explore and structure unstructured textual data (Rajman and Besançon 1998). These methods facilitate the identification of frequently used terms, thematic clusters and latent associations within the literature (Nalon et al. 2021). The integration of TM and TA enables the extraction of dominant research topics and the mapping of conceptual linkages between terms and themes. The increasing availability of digital databases such as Google Scholar, Scopus^®^ and Web of Science, along with advancements in bibliometric software tools, has significantly enhanced the capacity to conduct comprehensive literature analyses. TM and TA have been applied to animal science (Cesari et al. 2024; Trapanese et al. 2025) and veterinary science (Davies et al. 2024) domains but to our knowledge no studies have specifically utilized these techniques for health-related biomolecules in milk and dairy products. This review should help to identify gaps in knowledge that will inform future basic and translational studies that seek to build on the use of betaine and carnitine for animal production and human health outcomes.

## Materials and methods

### Dataset

A literature search protocol was established using Scopus^®^ to identify peer-reviewed scientific publications related to biomolecules in milk and dairy products, specifically focusing on the carnitine and betaine families. Carried out in February 2025, the search was refined according to the following criteria:

- Publication period: January 1970 to December 2024;
- Scientific fields: Agricultural and Biological Sciences, Biochemistry, Genetics and Molecular Biology, Medicine, Veterinary Science, Immunology and Microbiology, Chemistry, Pharmacology, Toxicology and Pharmaceutics and Health Professions;
- Document types: review articles, original research articles, book chapters, technical notes and conference papers.

**Fig. 1:**
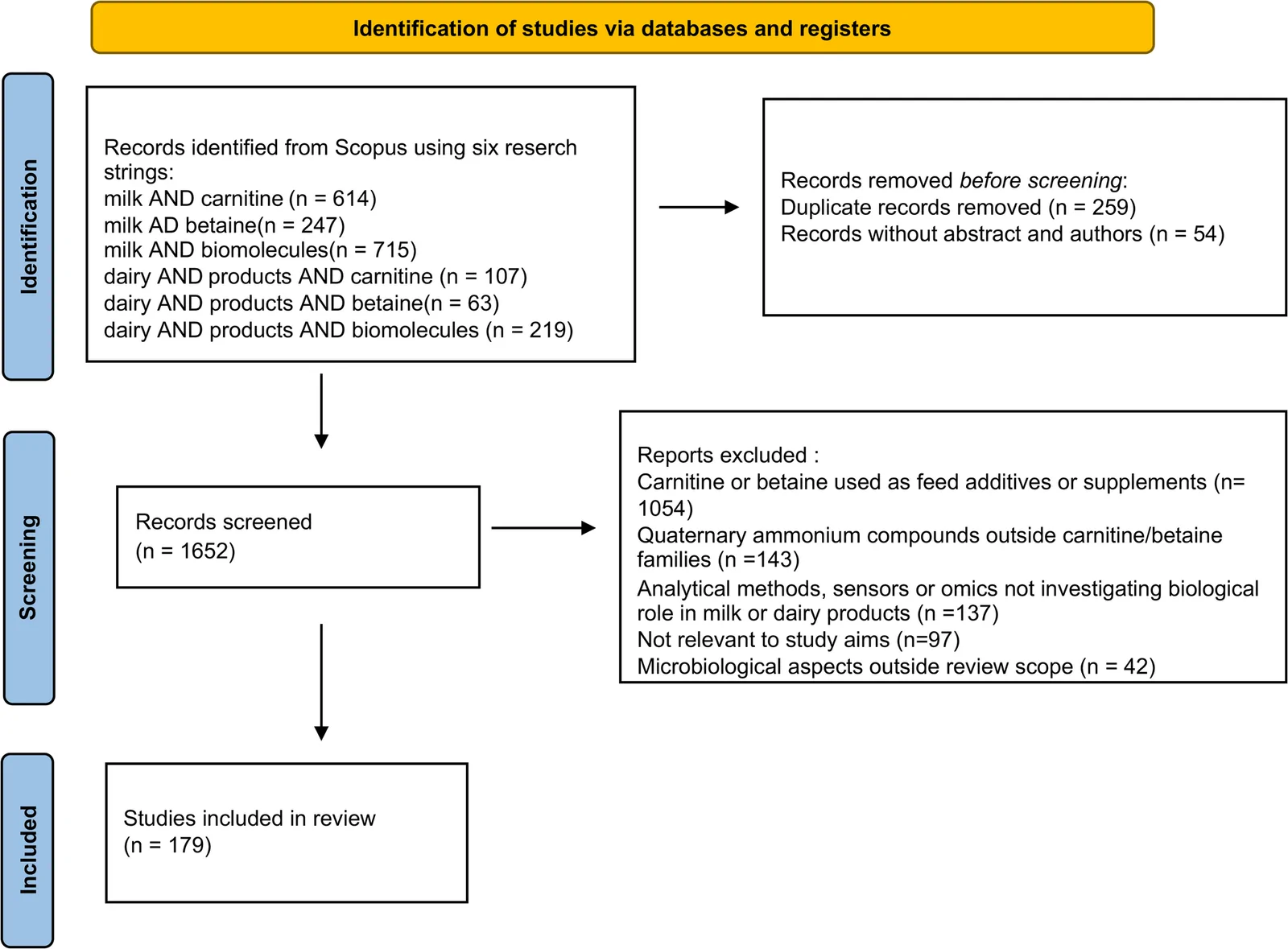
PRISMA flow diagram illustrating the study selection process, including identification, screening, eligibility assessment, and final inclusion of records in the review

The review was carried out according the guidelines of Preferred Reporting Items for Systematic reviews and Meta-Analyses (PRISMA) (Page et al. 2021) statement. Six search strings were employed (Fig. 1) to identify papers, resulting in 1,965 retrieved publications.

The records were organized in an electronic Excel workbook (Microsoft Excel^®^, v16.0) as described in Trapanese et al. (2024, 2025). Following data collection, preprocessing was performed to prepare the dataset for further analysis. This involved:

- Eliminating duplicates (259 papers) to account for records appearing in multiple search results;
- Removing incomplete entries including those missing abstracts (50 papers) and authors (4 papers);
- Screening for eligibility: For the purpose of this review, the term “carnitine family” included L-carnitine together with its naturally occurring acyl derivatives (e.g., acetyl-L-carnitine, propionyl-L-carnitine, butyrylcarnitine, and other short-, medium-, and long-chain acylcarnitines) whenever they were investigated as endogenous constituents of milk or dairy products. The “betaine family” comprised trimethylglycine (glycine betaine), γ-butyrobetaine, δ-valerobetaine, 5-aminovaleric acid betaine (5-AVAB) and other naturally occurring betaine derivatives identified in milk or dairy matrices. Screening for eligibility was independently performed by two researchers (A.S. and L.T.). Records were excluded when they: (i) investigated carnitine or betaine exclusively as feed additives or nutritional supplements (*n* = 1,054); (ii) focused on quaternary ammonium compounds outside the carnitine and betaine families (*n* = 143); (iii) addressed analytical methods, sensors or omics approaches without investigating the biological role of these compounds in milk or dairy products (*n* = 137); (iv) did not investigate the role, occurrence, or biological relevance of carnitine and betaine in milk or dairy products (*n* = 97); (v) focused exclusively on microbial characterization or fermentation processes without assessing carnitine or betaine-related biological aspects (*n* = 42). The process output is presented in the PRISMA flow diagram, and the corresponding workflow is provided in Fig. 1.

After completing the screening phase, 179 records were retained for analysis. Once the dataset was finalized, the authors affiliation, the publication year, journal, and country of publication, were used to profile and describe the dataset. The geographical affiliation for each paper was determined based on the country of the corresponding author. Descriptive statistics for publication year, country of publication, and record type, were calculated to profile the scientific corpus using Excel Pivot Tables and graphics.

### Text mining

The abstracts of the collected papers were compiled into a separate Excel spreadsheet with two columns: “*progressive ID*” and “*abstract*”. This spreadsheet was then prepared for TM analysis. All subsequent steps were carried out in the RStudio environment (R Core Team 2021), using functions from the packages “tm”, “snowballC”, “ggplot2”, “dplyr”, and “tidyverse”. In line with Sebastiani et al. preprocessing guidelines, the corpus underwent several preprocessing steps (Sebastiani 2002). Given the presence of both American and British English spellings, the authors standardized the corpus to American English. The preprocessing steps included tokenization, stop-word removal and stemming-lemmatization. The aim of tokenization was to break the text into meaningful units called tokens (Uysal and Gunal 2014).

The tokenization process specifically involved:

- Converting all words to lower case;
- Replacing escape symbols and special characters (e.g., “@”, “/”, “*”) with white spaces;
- Excluding certain characters, including punctuation, blanks and numbers.

The elimination of stop words was intended to remove terms that were either included in the research string or conveyed irrelevant information about the document’s content, such as common articles and conjunctions. The following words were excluded: “milk”, “dairy”, “products”, “biomolecules”, “carnitine”, “betaine”, “aim”, “group”, “%”, “vs”, “groups”, “significative”, “observations”, “observation”, “significant”, “significantly”, “mean”, “lcarnitin”, “method”, “can”, “research”, “measure”, “select”. The removal of those terms and their lexical variants was intentionally performed as part of the preprocessing workflow. Since these terms constituted the inclusion criteria and were therefore present in nearly all retrieved abstracts, retaining them would have artificially dominated the document-term matrix and reduced the ability of the Latent Dirichlet Allocation (LDA) algorithm to identify latent thematic structures. Their removal allowed the model to cluster documents according to the contextual biological, nutritional and technological concepts associated with these compounds, rather than on the ubiquitous presence of the search keywords themselves. This preprocessing strategy is consistent with standard practices in TM and TA, where query terms are commonly removed to improve topic discrimination (Contiero et al. 2019; Zuliani et al. 2021). The final step of the preprocessing phase consisted of applying stemming and lemmatization algorithms. Both procedures aimed to reduce words to their base or root forms, preserving the core meaning by removing suffixes or prefixes. The main difference between them is that stemming can generate non-words or partial words, whereas lemmatization returns words in their dictionary forms (Matuszewski 2023).

Following preprocessing, the corpus was manually inspected to verify the consistency of tokenization ensuring that chemically relevant terms, including hyphenated compound names and standard abbreviations, were correctly preserved before constructing the Document-Term Matrix (DTM). To examine the text content in depth, a DTM was created by setting the minimum number of letters to include to three, with documents as rows and terms as columns, displaying the frequency of each term in each document (Salton and Buckley 1988). The Term Frequency–Inverse Document Frequency (TF-IDF) method was applied to the DTM to assign specific weights to each term. This weight depended on the term frequency within a given document and its distribution across the entire set of records. This method ensured that each term relevance was based not only on its frequency within a document but also on its uniqueness and importance within the overall dataset. A TF-IDF value threshold equal or greater than 1.77 was used to construct a histogram of the most frequent words. Additionally, word cloud representation was made using the R package ‘wordcloud’, setting a minimum TF-IDF threshold of 0.5. The size of each word in the word cloud reflected its TF-IDF value, with larger sizes indicating higher values and thus emphasizing the most informative words. Associations among words with TF-IDF ≥ 1.77 were also examined. Associations were identified by measuring how often two words co-occurred. An association value close to 1 indicated that the two words frequently appeared together within the documents, whereas values near 0 indicated limited co-occurrence. Only associations ≥ 0.45 were considered in this paper.

### Topic analysis

The objective of the Topic analysis was to examine and interpret the content of a corpus of documents, extracting relevant insights from unstructured textual data. To achieve this, the Latent Dirichlet Allocation (LDA) technique was applied to the DTM. The LDA is a generative statistical model that assumes the presence of “K” hidden Topics distributed across a set of documents (Srivastava and Sahami 2009). The modeling process was carried out using the R package “topicmodels”, employing the Gibbs sampling method for parameter estimation (Grün et al. 2011). Since the optimal number of Topics is not typically known in advance, models were tested using various topic counts and the outputs were compared. To facilitate interpretation, the most characteristic words for each Topic were visualized using bar charts derived from the Topics beta values that represent the estimated probabilities that words are associated with a particular Topic. Each Topic was then labeled based on commonly accepted terminology in existing literature, with consensus among the research team. Finally, attention was given to identifying temporal patterns in the prevalence of each Topic. This involved generating a pivot table that tracked the yearly frequency of documents associated with each Topic. These temporal data were modeled using exponential regression and the goodness of fit was measured by the coefficient of determination (R²). A statistically significant trend was defined as one with *p* < 0.05, indicating meaningful changes in Topic prevalence over time. The analysis also identified which countries most frequently appeared in connection with each Topic, providing a geographic dimension of thematic distributions.

## Results

### Descriptive statistics

**Fig. 2:**
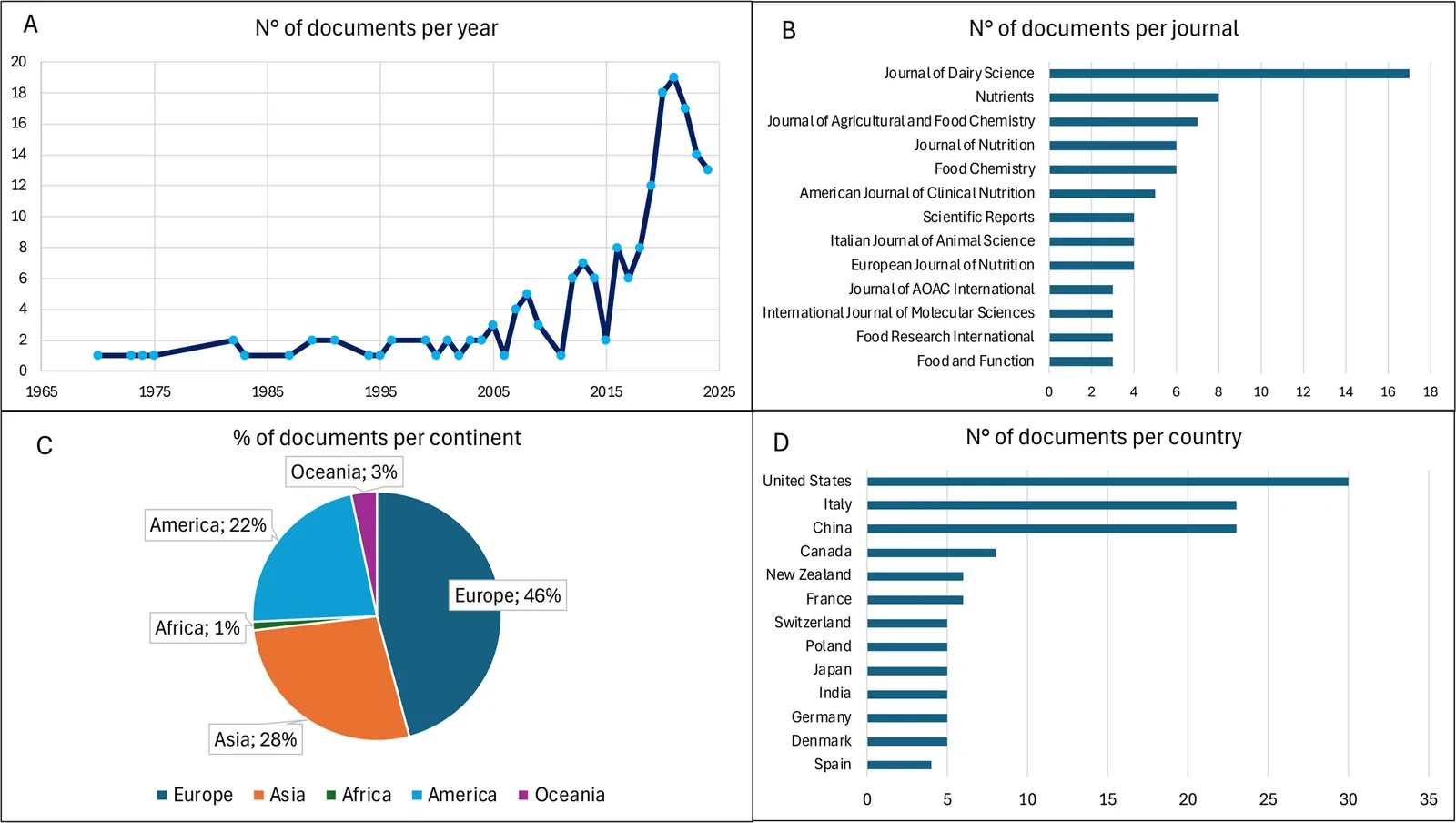
Descriptive statistics of dataset. (**A**) Shows the number of papers published from 1970 to 2024. (**B**) Represents the number of papers published per journal (at least 3 papers). (**C**) Shows the number of documents per continent. Finally, (**D**) depicts the number of papers published for country of the corresponding author

The literature research identified 179 peer-reviewed papers. Figure 2A shows the temporal trend in the publication of these papers from 1970 to 2024. Scientific publications increased steadily until 2021 and then showed a slight decline. Research articles were the most common type, accounting for 78.7% (141/179) of the total, followed by reviews (28/179; 15.6%), book chapters. (5/179; 2.8%) and conference papers (3/179; 2.6%), with others representing less than 1%. The distribution of published papers by journal title (including those with at least three papers during the period considered) is presented in Fig. 2B. Journal of Dairy Science, Nutrients, Journal of Agricultural and Food Chemistry, Journal of Nutrition, and Food Chemistry, were the most representative journals with 17, 8, 7, 6 and 6 papers, respectively. Europe was the most productive continent in terms of papers (82), followed by Asia (49) and America (40) while Africa and Oceania produced less than 10 papers combined (Fig. 2C). Figure 2D illustrates the geographical distribution of the 179 scientific papers, categorized by the corresponding author country. United States had the most publications (30), followed by Italy and China with 23 each. New Zealand accounted for all records of Oceania (6) and for Africa only Algeria and South Africa contributed with 1 paper each.

### Text mining

**Fig. 3:**
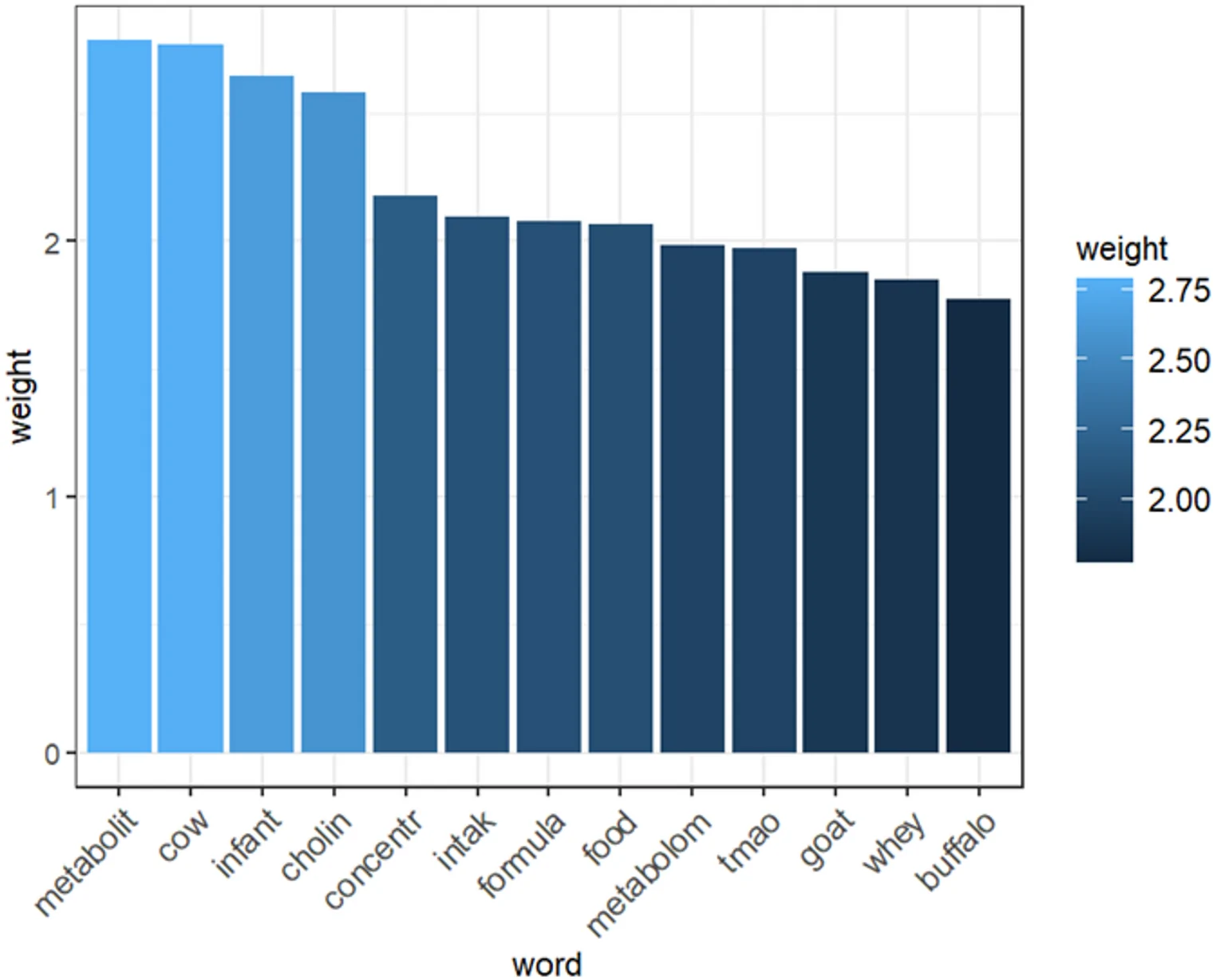
Histogram with the most frequent words (TF-IDF ≥ 1.77)

**Fig. 4:**
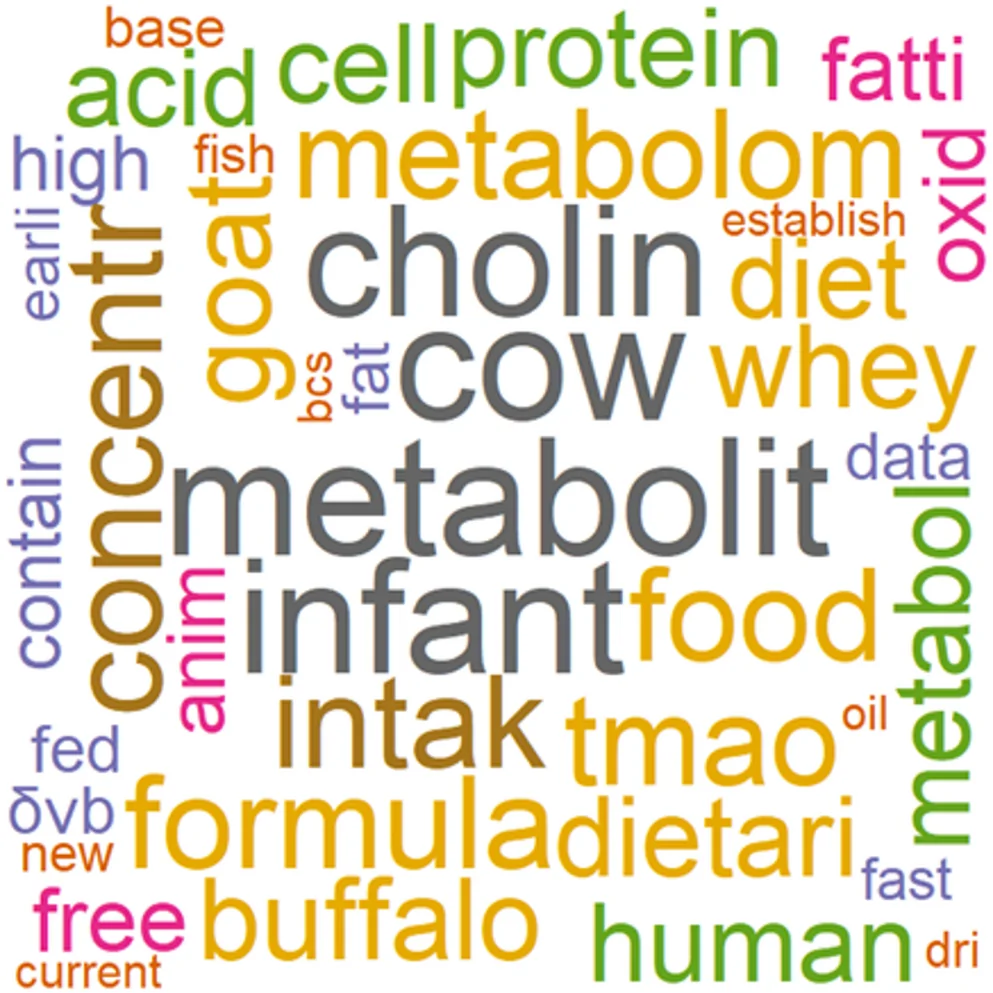
Word cloud representing the most frequent terms extracted from the corpus

After preprocessing the data and reducing sparsity, 1728 terms were retained from the initial set of 179 records. Figure 3 presents the most frequent words, each with a TF-IDF ≥ 1.77. The highest-scoring words were “metabolit” (2.78), “cow” (2.76), “infant” (2.64), “cholin” (2.57), “concentr” (2.17), “intak” (2.09), “formula” (2.07), “food” (2.06). Other words were below a score of 2: “metabolom” (1.98), “TMAO” (1.97), “goat” (1.88), “whey” (1.85) and “buffalo” (1.77). A word cloud is displayed in Fig. 4 where the size of each word reflects its frequency, with font size scaled according to its TF-IDF value. Word correlations are detailed in Table 1, which includes only words with a TF-IDF score ≥ 1.77 and a correlation coefficient of at least 0.45.

**Table 1:** Association about the most frequent words (TF-IDF ≥ 1.77) and the other words present in the documents analyzed. The analysis of word associations was based on stemmed and lemmatized terms

Word (TD-IDF ≥ 1.77)	Association (> 0.45)
Buffalo	Propionyllcarnitin (0.78), green (0.61), viabil (0.61), δvalerobetain (0.56), healthpromot (0.56), peroxid (0.56), bulk (0.52), highglucos (0.52), tmr (0.51), mix (0.49), oxygen (0.48), reactive (0.48), antineoplast (0.47), biomolecul (0.47), potenc (0.47), butyrylcarnitin (0.46), intracellular (0.46), treat (0.45)
Choline	Born (0.52), interv (0.48), onecarbon (0.48), women (0.46)
Food	Consum (0.53), awar (0.51), chosen (0.46), function (0.46)
Formula	Powder (0.58), fraction (0.51), nitrogen (0.49), nonprotein (0.49), nucleotid (0.47)
Goat	Nonprotein (0.69), nitrogen (0.68), fraction (0.59), made (0.58), powder (0.49), constitu (0.46).
Infant	Breastf (0.58), formulaf (0.56)
Intak	Women (0.48)
Metabolit	Analysi (0.54)
Metabolom	Nmrbase (0.52), nmr (0.51)
TMAO	Trimethylamine (0.62), noxid (0.55), cardiovascular (0.54), yolk (0.52), egg (0.48), atherosclerosi (0.47), gut (0.47), proatherogen (0.46)
Whey	Encapsul (0.55), author (0.49), broad (0.47), Chap. (0.47), wide (0.45)

### Topic analysis

**Fig. 5:**
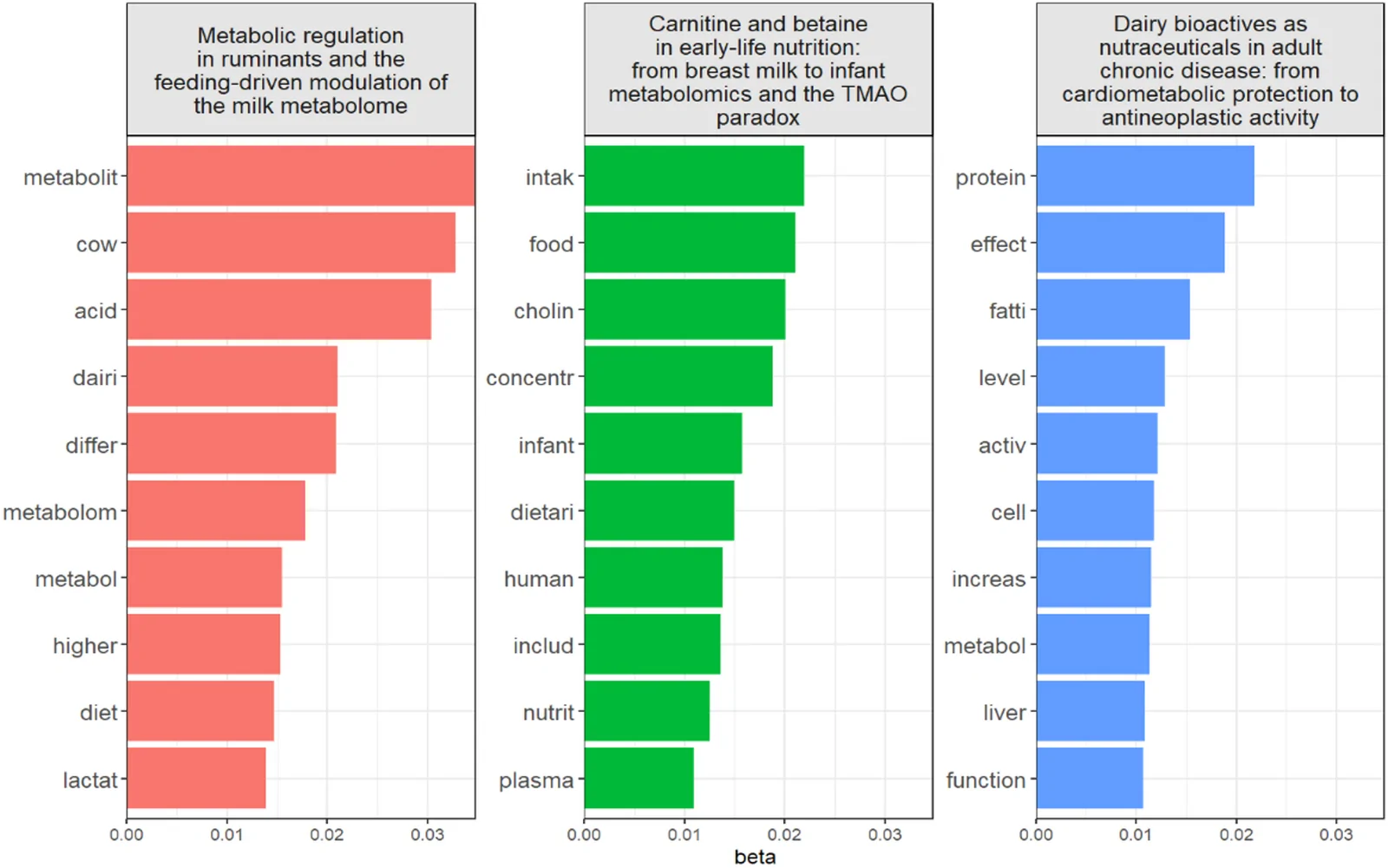
Result of Topic analysis. For each Topic the 10 most frequent words were shown. Beta value denoted the relative probability of each term belonging to that Topic

The Topic analysis identified three distinct Topics, each assigned a meaningful name based on the most frequent words and the content of associated articles. Figure 5 presents a histogram highlighting the top 10 words for each Topic. The most prominent Topic was Topic 2 (Carnitine and betaine in early-life nutrition: from breast milk to infant metabolomics and the TMAO paradox) encompassing 63 records (35%), followed closely by Topic 3 (Dairy bioactives as nutraceuticals in adult chronic disease: from cardiometabolic protection to antineoplastic activity) with 59 documents (33%), then Topic 1 (Metabolic regulation in ruminants and the feeding-driven modulation of the milk metabolome) with 57 papers (32%).

A trend analysis, covering the period from 1970 to 2024, is summarized in Table 2. Topic 1 contained the earliest publications, dating back to 1970, followed by Topic 3 (1973) and Topic 2 (1982). Topics 1 and 2 exhibited a growing trend over time, suggesting an overall exponential pattern in publications, whereas Topic 3 showed a slowdown. The distribution of publications across countries showed that different research priorities emerged among the leading contributors (Table 3). China predominantly contributed to studies investigating metabolic regulation in ruminants (Topic 1), whereas the United States was more represented by studies related to infant nutrition and metabolomic profiling (Topic 2). Italy showed a greater contribution to research on nutraceutical properties and health-promoting effects (Topic 3). These differences likely reflect the scientific expertise and research priorities of the respective countries. However, because this study was not designed as a bibliometric analysis, no causal relationship between geographical origin and research focus can be inferred.

**Table 2:** The table reports the first year of publication, the trend over time for each Topic

Topic	Name	Year of first publication	*R* ^2^	*P*-value
1	Metabolic regulation in ruminants and the feeding-driven modulation of the milk metabolome	1970	0.40	*p* < 0.001
2	Carnitine and betaine in early-life nutrition: from breast milk to infant metabolomics and the TMAO paradox	1982	0.53	*p* < 0.001
3	Nutraceutical properties and health-promoting effects of milk and dairy products	1973	0.33	*p* < 0.005

**Table 3:** Distribution of publications across the three identified topics according to the country of the corresponding author (Only first 4 countries)

Topic	Country (N^o^ of papers per topic)
Metabolic regulation in ruminants and the feeding-driven modulation of the milk metabolome	China (11)
	Italy (9)
	United States (7)
	Canada (5)
Carnitine and betaine in early-life nutrition: from breast milk to infant metabolomics and the TMAO paradox	United States (15)
	Japan (4)
	China (3)
	Germany (3)
Dairy bioactives as nutraceuticals in adult chronic disease: from cardiometabolic protection to antineoplastic activity	Italy (13)
	China (9)
	United States (8)
	France (3)

## Discussion

 A systematic analysis of the literature on biomolecules in milk and dairy products over the past 50 years was undertaken using Text Mining (TM) and Topic Analysis (TA) techniques. The approach sought to capture foods that contain compounds which supply energy and additionally provide beneficial effects for human and animal health, largely through antioxidant and anti-inflammatory properties (Zhao et al. 2018; D’Onofrio et al. 2021; Craig 2004; Bach 1982). The focus was on the carnitine and betaine families. It emerged that the study of the biochemical profile of milk and dairy products is an important field of research in China, USA and Italy. The European region would appear to have a particular interest in the nutraceutical quality of milk and dairy products and has generated a relatively large proportion (46%) of publications. The most recurrent words (TF-IDF > 2.5) found in our research were “metabolit”, “cow”, “infant” and “cholin”. As carnitine and betaines are metabolites present in both milk and dairy products, the word “metabolit” was expected. The term “cow” represents the primary source of milk and dairy products considered in the studies, used particularly in relation to “infant”, which is the third most frequent term. “Cholin” frequently appears in TM analysis of studies on carnitine and betaine in milk because choline, betaine and carnitine are metabolically connected through one-carbon metabolism and methyl-group transfer pathways, such as lipid transport and methylation (Konstantinova et al. 2008).

Papers in Topic 1 (Metabolic regulation in ruminants and the feeding-driven modulation of the milk metabolome) report carnitine and betaine as sensitive biomarkers of energy balance (Erfle et al. 1970, 1974; Snoswell and Linzell 1975), one-carbon metabolism (Pacífico et al. 2020; Salzano et al. 2022; Kim et al. 2023; Neglia et al. 2023), and diet in lactating ruminants (Servillo et al. 2018; Zivkovic et al. 2021). Mechanistically, L-carnitine is indispensable for mitochondrialβ-oxidation of long-chain fatty acids (LCFAs) through the carnitine shuttle system (Antunes et al. 2022). In ruminants, LCFAs are activated to acyl-CoAs and converted to acylcarnitines by carnitine palmitoyltransferase 1 (CPT1), translocated across the inner mitochondrial membrane by carnitine-acylcarnitine translocase (CACT), and reconverted to acyl-CoAs by carnitine palmitoyltransferase 2 (CPT2) in the matrix for β-oxidation (Matera et al. 2003). This shuttle is rate-limited at CPT1 which integrates fatty acid oxidation with energy status. Its impairment during negative energy balance limits mitochondrial LCFA entry, promoting toxic acyl-CoA accumulation in liver and muscle (Ghaffari et al. 2019, 2021). When hepatic oxidative capacity is exceeded, incomplete oxidation and triglyceride accumulation may lead to fatty liver, ketosis and systemic inflammation (Ghaffari et al. 2019, 2021). Betaine acts both as an organic osmolyte and as a methyl donor in the betaine‑homocysteine S‑methyltransferase reaction, regenerating methionine and maintaining S‑adenosylmethionine levels for DNA, RNA and protein methylation (Craig 2004). In high‑producing cows under stress, betaine contributes to cellular volume regulation and hepatic protection (Salzano et al. 2024).

The studies in Topic 1 showed that rumen-derived acylcarnitine and betaines are not just passive metabolites but may exert antioxidant, anti-inflammatory and endothelial-protective effects, thus bridging the gap between animal metabolic status and potential human health benefits of milk and dairy products. Variation in milk carnitine and betaine is tightly linked to species, breed, feeding strategy, energetic demand (lactation stage, body condition) and rumen microbial activity (Erfle et al. 1970, 1974; Snoswell and Linzell 1975; Salzano et al. 2022; Buitenhuis et al. 2013; Caboni et al. 2019; Sundekilde et al. 2014; Woollard et al. 1999). Dietary factors are key regulators of betaine and carnitine. Studies comparing pasture vs. total mixed rations in cows (Magan et al. 2019), green feed vs. conventional diets in buffalo (Salzano et al. 2021; Neglia et al. 2023), or moderate vs. high-grain diets showed that carnitine, betaine and their derivatives respond to alterations in fermentation profile, rumen health and systemic metabolic stress (Salzano et al. 2024). Pacífico et al. (2020) reported that high-grain feeding and the associated subacute ruminal acidosis are often linked to broader perturbations in the milk metabolome. Carnitine and betaine clustered alongside indicators of lipolysis, oxidative stress and inflammatory tone. Fresh forage and “green feeding” strategies tended to be associated with more favourable profiles including higher levels of rumen-derived bioactives and potentially more protective carnitine/betaine patterns in milk and dairy products (Salzano et al. 2021; Neglia et al. 2023). The inclusion of green forage in the diet of buffaloes was associated with distinctive changes in the ruminal metabolome (Neglia et al. 2023), transcriptome (Salzano et al. 2023), and microbiome (Salzano et al. 2024). These changes could be due to the higher amount of simple sugars that are able to improve rumen efficiency and nutrient utilization (Mordenti et al. 2021). The administration of simple sugars influences the profile of carbohydrate-hydrolyzing enzymes (CAZymes) in the rumen (Salzano et al. 2024). These enzymes are produced by ruminal microbes and are responsible for the hydrolysis of polysaccharides. Among them, glycosyltransferases is a vast family of enzymes that create glycosidic bonds, transferring sugar units from activated sugar donors to acceptor molecules (proteins, lipids, or other sugars). These enzymes are highly specific, as their residues can selectively modify the appropriate hydroxyl group on an acceptor molecule that contains many equally reactive hydroxyls (e.g. complex oligosaccharides) (Varki 2017). This process enables the release of fermentable sugars and the production of precursors essential for complex metabolic pathways, including the biosynthesis of functional biomolecules. Others papers in Topic 1 focused specifically on transition cows and body condition score (BCS), reinforcing the role of carnitine, betaine and related metabolites as markers of metabolic stress and adaptive capacity (Ghaffari et al. 2019, 2021; Dervishi et al. 2012). Cows with higher BCS at calving, or more severe negative energy balance, often show altered carnitine and acylcarnitine profiles consistent with increased mobilization and incomplete oxidation of non-esterified fatty acids (Ghaffari et al. 2019, 2021). Similarly, betaine and rumen-derived betaine analogues may change in ways that reflect osmotic and methylation demands during metabolic challenge and liver fat infiltration (Servillo et al. 2018; Pacífico et al. 2020). Some studies linked these metabolites to endothelial function and inflammatory signalling, highlighting that the metabolic stress of early lactation involves not only energy balance but also vascular and immunometabolic challenges (Salzano et al. 2022). This supported the interpretation that higher or lower milk carnitine and betaine concentrations in transition cows were not merely compositional traits, but integrative biomarkers of systemic metabolic resilience or vulnerability.

From a technological standpoint, carnitine and betaine are increasingly considered part of the “functional metabolome” of dairy products: they contribute to the nutritional profile and may also serve as markers of authenticity, feeding system (e.g. pasture-based vs. total mixed ration), and product quality. Some papers reported that various carnitine and betaine derivatives, including δ-valerobetaine (δVB) and specific acylcarnitines, are retained during processing and that their levels are influenced by the origin of the raw milk and may correlate with microbial biodiversity and product typology (e.g. mozzarella, ricotta, whey) (Salzano et al. 2019, 2022, 2024). Associations between δVB, acylcarnitines and cardiometabolic protection have also been proposed, suggesting that milk and cheese from specific production systems may deliver bioactive molecules with potential health benefits (Servillo et al. 2018; Salzano et al. 2022; Neglia et al. 2023).

Topic 2 (Carnitine and betaine in early-life nutrition: from breast milk to infant metabolomics and the TMAO paradox) collected 63 documents. Uniquely among the three Topics, Topic 2 focuses on the neonatal and early developmental period, during which carnitine and betaine shift from optional dietary components to conditionally essential nutrients whose adequate supply via milk critically determines metabolic and epigenetic programming outcomes. Topic 2 occupied the intersection of metabolically essential early-life nutrition and long-term health risks mediated by the gut microbiota, with milk and dairy products as central dietary sources. Early studies in Topic 2 established the biochemical indispensability of carnitine for mitochondrial β-oxidation of long-chain fatty acids in neonates and infants (Bach 1982; Borum 1995; Penn et al. 1987). “Infant”, a recurrent word of Topic 2, is associated with “Breastf” (0.58) and “formulaf “(0.56) highlighting that most of the documents in this Topic focused on infant nutrition. The collective message is that endogenous synthesis of carnitine is limited in early life, making dietary sources and in particular milk critically important. Clinical observations in infants with impaired carnitine status suggested altered fatty acid oxidation, hypoglycaemia and impaired energy homeostasis, supporting the concept of carnitine as a “conditionally essential” nutrient during specific developmental periods (Bach 1982; Penn et al. 1987; Sandor et al. 1982). Subsequent observational and interventional studies extended this framework by comparing breast-fed and formula-fed infants, assessing both dietary intake of carnitine and circulating concentrations (Bene et al. 2013; Brink et al. 2020). Infant formulas were often deficient or variable in carnitine and other methyl donors, prompting fortification strategies and regulatory oversight (Crill and Helms 2007; Gill et al. 2016). Formula fortification with carnitine can normalize plasma levels and support fatty acid oxidation but may not fully replicate the complex metabolite profile and bioactive matrix of human milk (Bene et al. 2013; Brink et al. 2020). Lombard et al. (1989) reported that children who consumed a vegetarian diet had lower levels of carnitine in both blood and urine compared to children who consumed a mixed diet or a lacto-ovo-vegetarian diet. Overall, the studies in Topic 2 indicate that human milk provides physiologically appropriate amounts and forms of carnitine, often balanced with other lipid-related metabolites (Bakshi et al. 2023; Gay et al. 2018). Carnitine should not be considered in isolation as its bioavailability and functional impact depend on the broader protein and methyl-donor context of milk. Betaine appeared less often in Topic 2 as a primary endpoint in early-life trials but is implicitly involved through its role as an osmolyte and methyl donor in one-carbon metabolism. This is linked to choline metabolism and homocysteine re-methylation (Konstantinova et al. 2008). The intake of methyl-donor-rich foods, including milk and dairy products, influences maternal plasma concentrations of choline, betaine and carnitine, which in turn are reflected, though not linearly, in human milk composition (Rafiq et al. 2022). Rafiq et al. (2022) reported that the concentration of metabolite was influenced also by non-dietary factors (ethnicity, maternal age, gestational age, pre-pregnancy, body mass index, physical activity and smoking history). There is considerable inter-individual variability in milk metabolite profiles within and across populations which reflects the complex interrelationships of genetics, diet, microbiota and lactation physiology (Gay et al. 2018). Notwithstanding individual variability in maternal consumption of dairy, meat, eggs and fish contributes significantly to circulating and milk levels of choline and betaine (Konstantinova et al. 2008; Rafiq et al. 2022). Variation in milk methyl-donor profiles could be related to infant growth trajectories, body composition and neurodevelopmental outcomes, though causality remains uncertain (Brink et al. 2020; Mygind et al. 2013). The body of information in papers of Topic 2 supports the view that human milk is not simply richer or poorer in specific nutrients, but is qualitatively different in how carnitine, betaine and related metabolites are packaged within the milk matrix and co-delivered with other bioactive compounds. Indeed, carnitines and betaines are part of a broader “methyl-donor milieu” in human milk, potentially shaping epigenetic programming, energy metabolism and microbiota development (Rafiq et al. 2022). A precautionary note is that most reports are observational without cause-and-effect relationships and the extent to which modifying maternal dairy intake or supplementing specific methyl donors can improve functional outcomes remains to be clearly established.

A second major conceptual pillar within Topic 2 is the Trimethylamine N-oxide (TMAO) paradigm. TMAO is a biomarker and potential mediator of cardiometabolic diseases and several reviews and cohort studies focused on how dietary carnitine, choline and betaine, partly derived from dairy products, are metabolized by the gut microbiota to trimethylamine (TMA) and subsequently converted by hepatic flavin monooxygenase (FMOs) to TMAO (Konstantinova et al. 2008; Brown and Hazen 2014; Rohrmann et al. 2016; Macpherson et al. 2020; He et al. 2021; Tacconi et al. 2023; Zheng and He 2022). Mechanistic and clinical studies indicate that elevated TMAO is associated with atherosclerosis, vascular inflammation, endothelial dysfunction and thrombotic risk, involving pathways such as altered cholesterol transport, activation of NF‑κB signalling and increased oxidative stress (Zhao et al. 2018; Brown and Hazen 2014; He et al. 2021; Tacconi et al. 2023; Zheng and He 2022). There would appear to be a paradox as the same molecules (carnitine, betaine and choline) that are indispensable in early life are implicated as substrates in potentially harmful TMAO pathways in adults. Other recent studies, however, provides evidence that lower plasma 5-amino valeric acid betaine (5-AVAB) levels are significantly associated with an increased risk of Type 2 Diabetes (T2D) (Wang et al. 2024). A study involving both children and adults found no association between plasma TMAO concentrations and adverse cardiometabolic or inflammatory outcomes, suggesting that in the healthy general population TMAO is not a direct marker of cardiovascular risk (Andraos et al. 2021). The studies in Topic 2 do not resolve this paradox, but collectively suggest that as with other nutrient intake, including carnitines and betaines, life stage should be considered since what is essential and beneficial during childhood may have a different risk–benefit profile in late adulthood. Nonetheless, the importance of the food matrix, (milk and dairy products), as source of methyl donors and nutrients (micronutrients, bioactive peptides, fermented products) that reduce net cardiometabolic risk, must be noted. These nutritional components relate to the composition of the microbiota which, differently shaped by nutrition at various stages of life, is a double-edged actor for the production of TMAO (Gay et al. 2018; Brown and Hazen 2014).

Topic 3 (Dairy bioactives as nutraceuticals in adult chronic disease: from cardiometabolic protection to antineoplastic activity) comprised 59 documents. This Topic focuses on the pharmacologically active dimension of the dairy metabolome in adults, framing milk bioactives (carnitine, betaine, whey-derived peptides, and fermented dairy fractions) as preventive or adjunct tools against chronic noncommunicable diseases. Topic 3 was centered around human-health and translational research. It presented milk bioactives, including carnitine and betaine-related metabolites, as preventive or adjunct tools for chronic diseases, rather than solely as markers of animal metabolism. Many papers in this Topic emphasized that specific sequences released from casein and whey proteins (through gastrointestinal digestion, fermentation, or enzymatic hydrolysis) can exert antioxidant, anti-inflammatory, antimicrobial, antihypertensive and immunomodulatory effects (Tidona et al. 2009; Naeem et al. 2022; Rakhra et al. 2020). Several papers focused on whey proteins and whey-derived fractions, including α-lactalbumin, β-lactoglobulin and whey protein concentrates/isolates, highlighting anti-cancer, immunomodulatory and metabolic effects (Cacciola et al. 2022, 2023). Recent preclinical research supports the chemopreventive and potential antitumor role of delactosed buffalo milk whey (DMW) in colorectal cancer (CRC) (Cacciola et al. 2023). In murine models of azoxymethane (AOM)-induced CRC, pretreatment with DMW significantly reduced both the number of early precancerous lesions and tumour incidence (Cacciola et al. 2023). Mechanistically, DMW restored the gut microbiota disrupted by carcinogen exposure, decreasing harmful and increasing beneficial bacterial genera. In addition, DMW reduced Helicobacter in AOM-treated mice, consistent with a shift toward a gut microbial profile less supportive of inflammation and CRC progression (Cacciola et al. 2023). Circulating cancer markers, such as butyric acid, 5-methylcytidine, and glycerophosphocholine, were also reduced following DMW administration. Furthermore, DMW induced cytotoxicity in human CRC cell lines by triggering apoptotic signalling cascades, including caspase-3 and PARP cleavage (Cacciola et al. 2023). These findings suggested that components of buffalo whey can exert direct antineoplastic effects and modulate the gut environment to promote intestinal health, supporting the potential of DMW as a dietary supplement to reduce CRC risk in humans. In a complementary in vivo study, whey from Mediterranean buffalo milk demonstrated anticancer activities in HCT116 xenograft mouse models of CRC. Although overall tumour growth did not differ markedly between treated and control groups, DMW administration resulted in distinct histopathological features of cell death within tumour tissues, consistent with both apoptotic and necroptotic mechanisms (Cacciola et al. 2022). Biochemically, these effects were associated with downregulation of proliferation markers (c-myc, phospho-Histone H3, p-ERK) and upregulation of cell death mediators (RIPK1, RIPK3, MLKL, cleaved caspase-3, PARP-1). Additionally, DMW promoted the expression of SIRT3, SIRT6, and PPAR-γ, while suppressing LDHA and PPAR-α, indicating multifaceted modulation of cancer cell metabolism (Cacciola et al. 2022). These new lines of evidence reinforce the promise of food-derived biomolecules, such as those from buffalo whey, not only for chemoprevention but also as potential adjuvants in CRC therapy, opening avenues for future translational studies and clinical applications. The above literature supported the view of whey not as a by-product, but as a concentrated source of nutraceutical molecules, including peptides and sulfur-containing amino acids. A substantial part of Topic 3 is devoted to fermented dairy products (yogurt, kefir, fermented milk drinks, functional beverages) and their microbiota-dependent bioactivity. This includes reviews on symbiotic products (probiotics + prebiotics) and whey-based functional beverages summarizing their possible roles in cardiometabolic health, immune function and gut (Hati and Prajapati 2022; Saadi et al. 2024). Fermented dairy can be considered “enhanced functional matrices” where the content and bioavailability of carnitine, betaine and related metabolites may be altered. Throughout Topic 3 milk and dairy nutraceuticals are tested in a wide range of chronic disease contexts (Table 4).

**Table 4:** Overview of chronic diseases and related evidence on carnitine and betaine compounds in milk and dairy products

Chronic diseases	Description	References
Metabolic disorders: Obesity, NAFDL, insulin resistance, diabetes, metabolic syndrome	Bioactive peptides derived from whey, milk and fermented dairy products exhibit antioxidative, anti-inflammatory, and metabolic regulatory functions, contributing to reduced risk of obesity, hypertension, and type 2 diabetes.	(AlNafea and Korish 2021; Aqib et al. 2019; Chen et al. 2014; Korhonen and Marnila 2013; Luo et al. 2024; Rosa et al. 2012)
Cardiovascular and endothelial dysfunction	δVB and betaine-rich dairy protecting endothelial cells, improving NO bioavailability, reducing ROS production and modulating inflammatory pathways	(Salzano et al. 2023; Martino et al. 2023)
Neurodegenerative problems	Acetyl-L-carnitine and other carnitine derivatives tested in Alzheimer’s disease and cognitive decline, often showing improved mitochondrial function, neurotransmitter balance and cognitive outcomes in animal and early clinical studies	(Kępka et al. 2022; Levine et al. 2005; Lioi et al. 2005)
Cancer	Donkey, buffalo whey and camel and milk fractions with anticancer and pro-apoptotic activity in various tumor cell lines; δVB and related betaines showing selective toxicity against colon cancer cells	(Tidona et al. 2009; D’Onofrio et al. 2021; AlNafea and Korish 2021)

Most papers in Table 4 showed that δVB is a constitutive metabolite in ruminant milk and dairy products, originating from rumen microbial metabolism and accumulating in milk and dairy products. In vitro models demonstrate that δVB‑rich dairy fractions protect endothelial cells by reducing oxidative stress, improving mitochondrial function, preserving nitric oxide bioavailability and attenuating inflammatory signalling (D’Onofrio et al. 2019; Salzano et al. 2019, 2021). In colon cancer models, δVB and related betaines inhibit tumour cell growth while sparing non‑tumorigenic cells, an effect linked to modulation of mitochondrial redox status and activation of sirtuin‑dependent metabolic reprogramming (D’Onofrio et al. 2020, 2021). Whey‑based interventions and δVB‑rich dairy matrices have been associated with improvements in endothelial mitochondrial function and redox status in models of diabetic vascular damage, partly via SIRT3‑dependent deacetylation of mitochondrial antioxidant enzymes such as superoxide dismutase 2 (SOD2) (D’Onofrio et al. 2021; Martino et al. 2023). In neurodegenerative settings, carnitine derivatives have been reported to support mitochondrial energetics and attenuate oxidative and apoptotic processes (Levine et al. 2005; Cristofano et al. 2016), although this literature is still limited and not always specific to dairy‑derived forms. This line of research provided a counter-narrative to the simplistic view of all trimethylated compounds from animal foods as harmful via TMAO. There is compelling evidence that milk-derived betaines are proposed as protective, modulating oxidative and inflammatory pathways, mitochondrial function and endothelial integrity (D’Onofrio et al. 2019; Salzano et al. 2019, 2021; Martino et al. 2023). Topic 3 hence supported the interpretation that milk betaines and carnitines are bioactive nutraceuticals, not merely metabolites. Another sub-cluster in Topic 3 examined non-bovine/buffalo milk (camel, donkey, goat) for disease-modifying potential. Camel milk is studied in models of diabetes, Non-Alcoholic Fatty Liver Disease (NAFLD), oxidative stress, and metabolic syndrome since camel milk peptides have been shown in mice to improve glycemic control through gut microbiota modulation (AlNafea and Korish 2021; Zhang et al. 2023). Donkey milk is described as a possible human milk substitute with anti-inflammatory and anticancer properties, particularly in models of colon carcinogenesis (Tidona et al. 2009; Li et al. 2020). These studies broaden the concept of dairy nutraceuticals beyond bovine milk, showing that different species provide distinct bioactive profiles that may be targeted to specific clinical situations (Aqib et al. 2019; Zhang et al. 2023; Li et al. 2020). Moreover, Topics 2 and 3 both address human health, they differ fundamentally in biological context and life stage and are therefore non-overlapping. Topic 2 frames carnitine and betaine as conditionally essential nutrients during the neonatal and early developmental period, a phase in which endogenous carnitine biosynthesis is limited and milk represents the critical exogenous source for mitochondrial β-oxidation, one-carbon metabolism, and early epigenetic programming. Topic 3, by contrast, addresses the translational and therapeutic potential of the dairy metabolome, including whey-derived fractions, fermented matrices, and species-specific milks in adult populations at risk for, or affected by, chronic non-communicable diseases. The TMAO paradigm, which appears across both Topics, functions as a conceptual bridge rather than a source of thematic overlap: it highlights how the risk-benefit balance of dairy-derived trimethylated compounds is life-stage-specific, being nutritionally indispensable in early life and context-dependent in adulthood.

The three topics identified by TM and TA delineate a mechanistic continuum: (i) from feed, rumen microbiota and animal metabolic status to the milk metabolome; (ii) from the milk metabolome to early‑life programming via carnitine and betaine‑rich human and bovine milk, with potential implications for mitochondrial function, one‑carbon metabolism and epigenetic regulation in the infant; and (iii) from lifelong dairy consumption to chronic disease risk or protection in adults, where the same molecules can feed into TMAO‑associated pathways but also deliver vascular and metabolic benefits within complex dairy matrices. This integrated perspective underscores both the potential and the complexity of exploiting milk‑derived carnitine and betaine as functional biomolecules in animal and human nutrition, health and wellbeing.

### Limitations

This systematic review has some limitations linked to the methodology adopted. Whilst synonymous terms were used during the initial search in Scopus^®^, the search was limited to the Elsevier database, excluding other sources such as Mendeley, Google Scholar and grey literature. This may have reduced the number of eligible papers. Also, only English-language articles with available abstracts were considered and this may have introduced a geographical and language bias, potentially underrepresenting research published in local journals or indexed in alternative databases. Therefore, although the identified thematic trends are likely to reflect the major directions of international research, they may not completely represent the global scientific landscape. From an initial pool of 1965 articles, only 179 were included based on expert opinion of those deemed most informative. Notwithstanding the limitations, this systematic review presents a comprehensive analysis of the literature related to carnitine and betaine in milk and dairy products. The review has identified the leading topics of scientific research and areas that require further basic and translational research.

## Conclusions

Carnitine and betaine in milk and dairy products represent a biologically relevant and translationally promising class of bioactives. Regulation of ruminant physiology through feeding strategies can be used to manipulate the synthesis of biomolecules with high nutritional importance. The available evidence indicates that milk-derived carnitines and betaines should be regarded not simply as compositional constituents, but as dynamic metabolic mediators linking animal physiology, dairy production practices, and human health and wellbeing. Their biological activity appears to be highly context-dependent, being influenced by developmental stage, food matrix, dietary patterns and gut microbiota composition. This complexity is exemplified by the TMAO pathway, in which metabolites that are indispensable during development early in life may exert different physiological effects later in life depending on metabolic and microbial context. Future research should focus on well-designed longitudinal, mechanistic, and intervention studies to elucidate how ruminant feeding strategies, dairy processing, species-specific milk composition, and gut microbiota, shape the bioavailability, metabolism, and biological effects of carnitine, betaine, δVB, and related metabolites. This knowledge will be essential to inform evidence-based nutritional recommendations and to design next-generation functional dairy products with targeted health-promoting and disease prevention and mitigation properties.

## Data Availability

The authors confirm that the data supporting the findings of this study are available upon request.
